# Clinical features and prognostic factors of ten patients with renal failure caused by IgG4-related retroperitoneal fibrosis

**DOI:** 10.18632/oncotarget.23103

**Published:** 2017-12-07

**Authors:** Wei Zhang, Feng Xue, Cui Wang, Leping Shao

**Affiliations:** ^1^ Department of Nephrology, The Affiliated Hospital of Qingdao University, Qingdao 266003, China; ^2^ Department of Anesthesiology, The Affiliated Hospital of Qingdao University, Qingdao 266003, China

**Keywords:** IgG4-related disease, retroperitoneal fibrosis, renal failure, prognostic factors, clinical features

## Abstract

IgG4-related retroperitoneal fibrosis (IgG4-RPF) is a newly recognized entity which often mimics cancer. We aimed to investigate the clinical features and the causes of renal failure, as well as to explore the factors affecting the prognosis of renal function by analysis of the clinical data of patients with IgG4-RPF. We reviewed clinical features of 10 patients with renal failure caused by IgG4-RPF, which was confirmed by pathology review and clinic-pathologic correlations. All patients were male, and the mean age at onset was 64.2 ± 10.0 years. Five patients were revealed with acute renal failure (ARF), while the other five ones with ARF on chronic kidney disease (CKD) (A on C) at diagnosis. Initial favorable responses obtained in 90% of the patients who underwent steroid therapy. The serum creatinine (SCr) level returned to normal in six patients including five with ARF and one with A on C, while those of the rest four patients with A on C restored to baseline levels (GFR remained below 60 ml/min/1.73 m^2^ however) after therapy. These four unrecovered patients had a history of CKD, a longer period of persistently elevated SCr, a thinner total renal parenchyma thickness, and continuous elevated serum IgG4 levels after steroid therapy, compared with those recovered patients (*P* < 0.05). We concluded that recovery and long-term prognosis of the disease were primarily associated with timely diagnosis and proper treatment.

## INTRODUCTION

IgG4-related disease (IgG4-RD) is a newly recognized entity characterized by tumour-like swelling in multiple sites caused by lymphoplasmacytic infiltration and sclerosis, and it has been associated with elevated serum IgG4 level and infiltration of IgG4-positive plasma cells in the involved organs and tissues [1, 2]. IgG4-RD, which can occur as a multi-organ entity, unifies a large number of medical diagnoses previously considered as being confined to a single organ system. This disorder was firstly described from autoimmune pancreatitis (AIP) [3, 4], which is now regarded as a pancreatic lesion of IgG4-RD [5, 6].

More and more reports on IgG4-RPF as an extrapancreatic lesion of AIP have been published recently, and to be more exact, IgG4-RPF is now regarded as a typical lesion associated with IgG4-RD [7, 8]. Previous studies have suggested that retroperitoneal fibrosis (RPF) is the most commonly associated condition outside of the pancreatobiliary system in patients with IgG4-related autoimmune pancreatitis [9, 10], however, only a few papers published on RPF have introduced the concept of IgG4-RPF [11, 12]. In 2012, a consensus statement on the pathology of IgG4-RD, focusing on histopathology, proposed the criteria for its diagnosis [5]. Serum IgG4 concentration elevations were once considered necessary for the final diagnosis, but normal serum IgG4 concentrations are now well described, even in the setting of active, biopsy-proven disease [13–15]. Careful analysis of imaging data and correlation with the clinical scenario is required to achieve a correct diagnosis. In this study, the diagnoses were confirmed by pathology review according to consensus diagnostic criteria and clinicopathologic correlations; but serum IgG4 concentrations were not necessary any more [13].

Similar to RPF, IgG4-RPF also develops around the abdominal aorta and iliac arteries, often entrapping the ureters and ultimately leading to hydronephrosis and renal failure^1^. Although the published literature included several small case series and individual case reports of IgG4-RPF, only very few reports focused on renal failure caused by IgG4-RPF, and the investigation of long-term clinical follow-up and prognostic factors of renal function were absent. We accordingly examined the clinicopathological features of patients with renal failure caused by IgG4-RPF, and investigated their prognostic factors of renal function.

## RESULTS

### Patient demographics

All of the ten patients were male. The mean age at onset was 64.2 ± 10.0 years old. The average time from the symptoms onset to the diagnosis being established was 10.6 ± 7.6 months. The mean duration of elevated SCr levels before steroid therapy was 1.5 (0.3–26.0) months. Various initial symptoms were predominantly related to RPF, including back pain (10%), edema of the lower extremities (20%), oliguria (10%), weight loss (20%) and hypertension (10%), while other initial symptoms were due to non-RPF associated diseases, including swelling of salivary glands (30%), mediastinal tumour (10%), swelling of the lacrimal glands (10%), dry mouth (10%), pulsatile abdominal mass (10%) and pain of the lower extremities (10%). Allergic history was found in five patients (50%). Past medical history included hypertension (3 cases), chronic kidney disease (CKD stage 3, 5 cases), coronary heart disease (1 case), cerebral haemorrhage (2 cases) and urinary bladder tumour (1 case) (Table 1).

**Table 1:** Clinical findings of patients with IgG4-related retroperitoneal fibrosis

Case	Age^1^	Initial symptoms	Dura-tion^2^	Allergic history	Past disease history
1	53	Swelling of salivary glands	12	Penicillin allergy, Rhinitis	Chronic kidney disease (eGFR = 39 ml/min/1.73 m^2^)
2	58	Mediastinal tumour	12	No	Hypertension
3	84	Acute renal failure	6	No	Hypertension, Coronary Heart Disease, Cerebral Infarction
4	76	Pulsatile abdominal mass, lower extremitypain	14	Asthma	Bladder Cancer,Cerebral haemorrhage Chronic kidney disease (eGFR= 38 ml/min/1.73 m^2)^
5	52	Lower extremity oedema, weight loss	5	No	-
6	65	Hypertension, weight loss	8	Rhinitis	-
7	70	Dry mouth	26	Penicillin allergy, Rhinitis	Chronic kidney disease (eGFR = 34 ml/min/1.73 m^2^) diabetes mellitus
8	61	Back pain, lower extremity edema	15	No	Hypertension, chronic kidney disease (eGFR = 31ml/min/1.73 m^2^)
9	63	Swelling of salivary glands and lacrimal glands	2	No	Chronic kidney disease (eGFR = 55 ml/min/1.73 m^2^)
10	60	Swelling of salivary glands	1	Rhinitis	-

^1^Age at onset (years); 2Time from onset of symptoms to the time of diagnosis (months); eGFR was calculated by CKD-EPI formula.

### Organ involvement

Evident lesions of IgG4-RD were present in other eleven organs besides retroperitoneum in this series of patients, including the renal interstitium (*n* = 1), mandibular gland (*n* = 5), lacrimal gland (*n* = 2), peripheral lymph nodes (*n* = 3), pancreas (*n* = 2), thymus (*n* = 1), gastrointestinal tract (n = 1), biliary system (*n* = 1), aorta abdominalis (*n* = 1), lung (*n* = 1) and inner ear (*n* = 1). The mean number of organs involved was 3.5 ± 1.3, which was not correlated with the serum IgG4 concentration before therapy (*r* = 0.142, *P* = 0.695).

### Laboratory examination at diagnosis

The median WBC count was 6.7 (3.9–26.6) × 10^9^/L, and leucocytosis was observed in 1 patient (10%); the mean eosinophil count was 1.9 ± 1.6 × 10^9^/L, without eosinophilia detected. The median C-reactive protein (CRP) level was 6.8 (0.6–42.3) mg/L, and high CRP level were seen in 6 patients (60%); all cases presented elevated ESR with an average level of 34.5 (17.0–110.0) mm/1 h. Positive ANAs were seen in 20% of patients, and 90% of patients (9 patients) showed elevated serum IgG4 with an average IgG4 level of 8.1 (1.4–13.1) g/L. 70% cases with hypocomplementemia and 90% patients with anemia resulted in a mean complement C3 of 0.8 ± 0.2 g/L and an average Hb of 90.5 ± 22.9 g/L, respectively. Five A on C cases had an average GFR of 39.4 ± 9.3 ml/min/1.73m^2^ and five ARF cases had a mean SCr of 632.5 ± 269.3 μmol/L. Four patients had mild urine protein and one patient was confirmed with IgG4-related tubulointerstitial nephritis (IgG4-TIN) by kidney biopsy. One of our patients developed septicaemia which suggested a severe inflammatory reaction in the course of disease.

All the four patients with proteinuria had hypocomplementemia with a median complement C3 level of 0.7 (0.7–0.8) g/L, while the patients without proteinuria (*n* = 6) had a higher median level of 0.9 (0.8–1.1) g/L, and there was borderline significant difference between the two groups (*P* = 0.057).

### Radiology

The retroperitoneal masses were detected primarily in the left renal hilus in 1 patient (Case 10), in the periaortic region in 5 patients (Case 2, 3, 4, 8 and 9), and in the periiliac area in 4 patients (Case 1, 5, 6 and 7). One case was complicated with abdominal aortic aneurysm (Case 4), and abdominal aortic calcification was found in 6 cases. The retroperitoneal masses involved the ureter and were associated with ureterohydronephrosis in all patients (left, *n* = 1; bilateral, *n* = 9). The ureter was involved in the retroperitoneal masses which led to ureterohydronephrosis in all patients. Renal atrophy was observed in 6 cases (Case 1, 4, 5, 7, 8 and 9), including one with bilateral renal atrophy and five with unilateral renal atrophy. The median total renal parenchymal thickness was 3.0 (1.7–3.9) cm. There was moderate correlations between the duration of elevated SCr levels before steroid therapy and total renal parenchymal thickness (1.5 cm (0.3–26) vs 3.0 cm (1.7–3.9), *r* = 0.75, *P* < 0.05).

Three patients (Case 2, 6, and 10) accepted FDG-PET test. FDG uptake was detected in the retroperitoneal masses in all the three patients and was also detected in many of the other IgG4-related lesions.

### Pathology

Abundant infiltration of IgG4-positive plasma cells, storiform fibrosis and obliterative phlebitis were histologically confirmed in the resected salivary glands (Case 1, 3, 7, 9 and 10), peripheral lymph nodes (Case 4 and 7), lacrimal gland (case 8), pancreas (Case 6), bile duct (Case 5), and thymus (Case 2), as well as needle biopsies including the organs of retroperitoneum (Case 8) and kidney (Case 2). We found a significant IgG4 + plasma cells infiltration, with a median number of IgG4 + plasma cells/high power field (hpf) of 50.5 (34–60), and the ratio of IgG4 + to IgG + was 63.5% (50–80%).

### Therapy

In the initial phase of clinical course, four patients were treated with surgical intervention, including implanting ureteral stents (Case 1, 2 and 8) and abdominal aortic stents (Case 4). Six patients accepted hemodialysis (Case 1, 2, 3, 6, 7 and 8; 60%). Steroid therapy was implemented on all patients, among whom 9 patients responded well. The initial dose of oral prednisolone was 0.6–0.8 mg/kg/d, which was gradually tapered by 2.5-5.0 mg every one to two weeks according to serological and imaging tests. Maintenance therapy with low-dose of prednisolone (5-10 mg/day) was administered in all of these patients. One patient (Case 9, 10%) was not sensitive to corticosteroids and received immunosuppressive treatment (Intravenous cyclophosphamide was initiated at 0.6 g/week with a total accumulated dose of 7.0 g .) along with low-dose corticosteroids. The whole course lasted for 6-8 months according to the function recovery of organs involved.

After steroid therapy, the median number of organs involved decreased to 1.0 (1.0–2.0), which was significantly less than pretherapy (*P* = 0.007); the median serum IgG4 concentration was 2.1 (1.2–11.0) g/L, which was also obviously decreased compared with those before treatment (*P* = 0.009). However, no correlation was observed between the measurements (*r* = 0.290, *P* = 0.416).

At the end of course of steroid therapy, SCr decreased significantly to 139.6 ± 76.7 μmol/L (*P* = 0.005), and six patients recovered to normal SCr level but four patients developed CKD later (Cases 1, 4, 7 and 9, 40%).

Four patients progressed to CKD (Case 1, 4, 7 and 9) with an average eGFR of 31.5 ± 11.2 ml/min/1.73m^2^, all of whom had a history of CKD and elevated SCr levels at diagnosis. In the univariate analysis, the patients who progressed to CKD were more likely to had a CKD history at diagnosis (100% vs 16.6%, *p* = 0.001), a longer duration of elevated SCr before therapy (12.8 ± 11.2 months vs 0.8 ± 0.6 months, *p* = 0.027), a thinner total renal parenchymal thickness (2.2 cm (1.7–2.3) vs 3.8 cm (2.5–3.9), *p* = 0.010) and higher post-treatment serum IgG4 concentrations (5.1 g/L (2.3–11.0) vs 1.3 g/L (1.2–2.3), *P* = 0.010). There were no significant differences in disease duration, age at onset, serum inflammatory markers, the treatment other than prednisolone (Table 2).

**Table 2:** Epidemiological, clinical and biological features of the ten patients with IgG4-related retroperitoneal fibrosis and their difference between the recovered and unrecovered groups in renal function

	Recovered patients with Normal SCr *n* = 6	Unrecovered patients with CKD *n* = 4	*p*-value
Onset age (years)	63.3 ± 11.0	65.5 ± 9.9	0.755^&^
Duration between onset and diagnosis (months)	7.8 ± 5.0	14.75 ± 9.7	0.347^&^
Total renal parenchymal thickness (cm)	3.8 (2.5–3.9)	2.2 (1.7–2.3)	0.010^▲^
Duration of elevated SCr level before steroid therapy (months)	0.8 ± 0.6	12.8 ± 11.2	0.027^&^
**Disease history**			
Hypertension	2/6	1/4	0.129^*^
Diabetes	0/6	1/4	0.305^*^
CKD	1/6	4/4	0.001^*^
Smoking	1/6	1/4	0.857^*^
Biological findings at diagnosis			
IgG4 (0.03–2.01 g/L)	7.3 ± 3.6	9.2 ± 5.2	0.563^&^
SCr (31–132 μmol/L)	660.5 ± 279.0	590.5 ± 289.8	0.712^&^
Hb (130–175 g/L)	95 (84–138)	71 (57–111)	0.171^▲^
ESR (<15 mm/1 h)	28.5 (17–97)	47.5 (23–110)	0.610^▲^
CRP (0–5 mg/L)	14.8 ± 15.1	4.1 ± 3.1	0.147^&^
Complement C3 (0.9–1.8 g/L)	0.8 ± 0.1	0.9 ± 0.2	0.591^&^
High level of serum IgG4	5/6	4/4	0.381^*^
Biological findings of post–treatment			
Serum IgG4 concentration	1.3 (1.2–2.3)	5.1 (2.3–11)	0.010^▲^
High level of serum IgG4	1/6	4/4	0.019^*^
SCr (31–132 μmol/L)	95 (73–128)	294.5 (179–330)	0.010^▲^
**Treatment**			
Favourable initial response to steroid therapy	6/6	3/4	0.305^*^
Hemodialysis	4/6	2/4	0.381^*^
Intraureteral stent insertion with a double J catheter	2/6	1/4	0.807^*^

^*^By Fisher's Exact Test (Categorical), ^&^Unpaired *T*-Test (Parametric), ^▲^Mann–Whitney *U* Test (Non-Parametric).

### Follow-up

All the patients were followed up for a median period of 48 (12–74) months. Over the entire follow-up, no patients died or developed tumours. Two patients (20%) suffered a relapse during follow-up. One patient developed sclerosing cholangitis after steroid withdrawal, and the other experienced sensorineural hearing loss (SNHL) during the course of steroid reduction. Restarting prednisolone at a dose of 40 mg/d led to a complete remission in both of them.

## DISCUSSION

IgG4-RPF is a rare disease which often mimics cancer, and has a high risk of developing CKD. There have been several reports on IgG4-RPF [12], but none of them were conducted from the aspect of renal function on the short and long-term outcomes, as well as prognosis risk factors. This study showed the importance of associating clinical, biological and radiological data with the diagnosis of IgG4-RPF and also suggested that an improving prognosis was related to steroid therapy. The originality of this study was that it illustrated the evolution of IgG4-RPF from obstructive kidney disease, which was usually managed by urologists, to an inflammatory disease better managed by early medical care with corticosteroids, and it explored the factors affecting the prognosis of renal function. However, a weak point to note was the small size of our study population, although it was still a respectable number considering the rare incidence of IgG4-RPF.

IgG4-RD is an immune-mediated fibro-inflammatory condition that can affect nearly any organ^1^. The manifestations of IgG4-RD in the retroperitoneum consist of retroperitoneal fibrosis and inflammatory abdominal aortic aneurysms. IgG4-RD occurs predominantly in men in their fifth and sixth decades of life^1^. The mean age at onset was 64.2 ± 10.0 years old in this study, similar to those in previous reports.

Several reports have suggested that concurrent RPF occurs in approximately 10% of cases of IgG4-RD [12, 16]. However, there are currently no data on the incidence of renal failure caused by IgG4-RD. In our study, concurrent RPF occurred in 13 of 36 patients (33.3%) with histologically diagnosed IgG4-RD, and 10 of 13 (77%) experienced renal failure.

IgG4-RD usually occurs with a history of chronic allergic conditions, such as allergic rhinitis, atopic eczema, asthma and peripheral blood eosinophilia [10, 11]. Some researchers believe that allergic rhinitis is one of clinical manifestations of IgG4-RD [17]. Five patients (Case 1, 4, 6, 7, and 10) had past allergic history in our study, but none of them was observed to have eosinophilia. It was ever reported that IgG4-RD presents in a subacute fashion [2]. The duration from the onset of symptoms to the diagnosis being established was 10.6 ± 7.6 months in this study. Abdominal and/or back pain was reported as the most common symptoms, affecting 88–94% of RPF patients [18–21]. However, in the present series, symptoms predominantly related to RPF (back pain and edema of the lower extremities) were observed in only 5 patients, and the remaining 5 patients were reported with initial symptoms due to other associated diseases, including swelling of salivary glands and lacrimal glands, mediastinal tumours and new onset diabetes. Five patients had a history of CKD (Case 1, 4, 7, 8 and 9), while the remainder had ARF at diagnosis. And the clinical presentations of IgG4-RPF are quite variable. In addition, the disease is not continuously active in most patients, and may range from organ-limited to multi-systemic forms, and the clinical manifestations can be synchronous or metachronous [2]. Thus, we should pay close attention to patients suspicious for IgG4-RD with ARF or CKD, and should carry out comprehensive inspection and assessment in order to prevent misdiagnosis and delay of treatment, and should pursue life-long follow-up.

The laboratory findings observed in the present series were similar to those previously reported in the literature, including increased levels of CRP (60% vs. 58–79% [20–22]) and positivity for ANA (20% vs. 5–42% [19, 20, 22]. Anemia was common in our cohort with the incidence rate of 90%, while 38% [19] –64% [21] has been reported in the literature; this disparity may be explained by a greater proportion of subjects with renal failure and renal anemia in this study.

Hypocomplementemia has also been frequently observed in IgG4-RD, and complement C3 levels commonly returned to normal after treatment [23–25]. However, the clinical significance and pathophysiology remain unclear. Kihara M et al found that a decrease in serum complement preceded deterioration of the disease, and clinical improvement was observed in accordance with normalization of serum complement in 4 patients [26]. Wallace ZS et al also found that patients with IgG4-RD had hypocomplementemia and elevated serum IgG4 concentrations, and those patients also tended to have higher concentrations of other serum IgG subclasses [13]. In our series, 7 cases had hypocomplementemia, one of them with the concomitant disease of IgG4-TIN; all the patients with proteinuria had hypocomplementemia. These findings suggested that serum complement may be one of the indicators of disease activity [26].

In this study, one patient in our centre accepted renal biopsy, and renal pathology confirmed IgG4-RPF combined with IgG4-TIN. As a matter of fact, the urinary system can be affected by IgG4-RD in a variety of patterns. Ureteral encasement and renal dysfunction are closely related to IgG4-RPF, while underlying tubulointerstitial nephritis and membranous glomerulonephritis can also be present [9, 12, 16, 18, 27]. A lesion of the arteries, IgG4-related plasma cell arteritis, has also been observed [28]. Additionally, the kidney can also be affected by extrarenal manifestations of IgG4-RD, including ureteral inflammatory pseudotumour [29–31]. Therefore, proteinuria and urinary sediment should always be checked, and we should note that RPF may be combined with IgG4-TIN, glomerular disease and even drug-induced nephrotoxicity, leading to renal failure, especially when unilateral obstruction or mild form of bilateral obstruction is not solely responsible for renal failure.

IgG4-RPF is often sensitive to steroid therapy, and remission occurs after therapy of 6-9 months [8]. The effects of steroid therapy have no relationship with whether iRPF is associated with IgG4-RD or not, as well as whether serum IgG4 levels are elevated or not [21]. Medical therapy should also be initiated as early as possible. Induction therapy of prednisone (0.6–0.8 mg/kg/d for the first month) is the best option to curb disease activity [32]. Satisfying effects with steroid therapy were achieved in 9 patients in this series, excluding only one patient (Case 9) without response. In our study, 3 patients (Cases 1, 2, and 8) with significant obstruction of the urinary tract underwent endoureteral interventions to relieve the obstruction as the initial treatment by urologists. All the 3 patients subsequently developed renal failure and required hemodialysis. Thus surgical treatment may only address the issue of ureteral obstruction, it does not prevent disease progression or recurrence, and has no effect on systemic manifestations. Further, the procedure of ureteral stents might be constricted by the severe fibrotic process around the ureter. Additionally, comparing patients with ureteral stents and without ureteral stents at any stage of steroid therapy, there was no significant difference between them. Therefore, steroid therapy might be superior to surgery in patients with ureteral obstruction, however, this conclusion requires more evidence to confirm. On the other hand, comparing patients complicated with proteinuria with those without proteinuria, there was no significant difference in SCr level at any point of steroid therapy. And steroid therapy has significant effects on every organ involved in IgG4-RD, independent of the number of organs involved [13].

The prognosis of iRPF is related to renal involvement. No predictors or risk factors have been identified for relapse of IgG4-RPF [33]. Risk factors for CKD have rarely been evaluated in the literature on iRPF. Gallais et al identified the ages of patients, the presence of diabetes and the initial value of SCr as factors predicting CKD [34]. Labidi J et al, in a recent study, identified high SCr , high urea, creatinine clearance < 60 mL/min at diagnosis and the use of ureteral stents as factors associated with the development of CKD [35]. In our cohort, all of the patients who developed chronic renal failure (CRF) had a history of CKD at diagnosis, longer periods of persistently elevated SCr, a thinner renal parenchyma thickness (Of note, the total renal parenchymal thickness was not an independent predicting factor since it had moderate correlations with the duration of elevated SCr levels before steroid therapy.), and continuously elevated serum IgG4 levels after steroid therapy, compared with the patients whose SCr returned to normal (*P* < 0.05). Patients with ARF had a better prognosis of renal function than those with CKD history during steroid therapy.

Therefore, recovery and prognosis of renal function were primarily related to timely diagnosis and treatment. In our centre, post-treatment SCr levels decreased to 139.6 ± 76.7 μmol/L, which was significantly lower than pretherapy, thus delaying the progression of CKD. In addition, the Case 8, who was complicated with CKD, achieved an approving recovery of renal function. Thus, patients with CKD caused by IgG4-RPF should carefully evaluate the disease activity, and actively try to use steroid therapy in order to restore the renal function to the maximum extent. Persistently elevated IgG4 concentrations after therapy may indicate poor prognosis of renal function; however it is still inconclusive whether serum IgG4 concentrations return to normal at the end of steroid treatment.

In conclusion, this center, for the first time in China, performed the analysis of clinical features and prognostic factors in 10 patients with renal failure caused by IgG4-related retroperitoneal fibrosis, hoping to provide information for nephrologist in the diagnosis, therapy and prognosis estimation. However, these results must be confirmed by a prospective, multicentre study with a larger number of patients. The diagnosis of and therapy for IgG4-RPF is not yet standardized, and further studies are needed to determine the optimal treatment and long-term follow-up outcomes for the patients with renal failure.

## MATERIALS AND METHODS

Between January 2001 and January 2015, a total of 36 patients treated at the Affiliated Hospital of Qingdao University were diagnosed with IgG4-RD, of which, 13 patients were with IgG4-RPF (33.3%) and 10 (77%) patients revealed renal failure caused by IgG4-RPF. Here, those 10 patients with renal failure were involved in our study. The study protocol was approved by the Ethics Committee of the Affiliated Hospital, Qingdao University. Informed consent was obtained from each subject.

The diagnosis of IgG4-RD was predicated upon the careful clinicopathologic correlation. Through observing biopsy materials from at least one organ of every patient, we concluded that the major histopathologic features included dense lymphoplasmacytic infiltration, fibrosis with a storiform pattern in focal (or diffuse) areas, and obliterative phlebitis. Significant IgG4+ plasma cell infiltration was considered if there were more than 10 IgG4+ plasma cells per high power field (hpf) and an IgG4+-to-IgG+ ratio of at least 50%. RPF was diagnosed by contrast-enhanced computed tomography (CT) following commonly accepted radiological criteria, including soft tissue masses surrounding the aorta and/or adjacent tissues on CT and/or magnetic resonance imaging (MRI). Patients were excluded if they had possible secondary causes of idiopathic retroperitoneal fibrosis, such as drugs (methysergide, ergotamine), malignancies, external beam radiation, tuberculosis, or asbestos exposure [36].

Renal parenchymal thickness was measured using the first and last noncontrast CT image levels from the anterior, posterior and lateral locations of the kidney that clearly included the collecting system. The mean of these measurements was defined as the renal parenchymal thickness. The sum of bilateral renal parenchymal thickness measurements was the total renal parenchymal thickness [37].

### Data analysis

Statistical analysis was performed using SPSS software, version 19 (Chicago, IL, USA). Significant differences for continuous, normally distributed data were analysed by Student *t*-test; all continuous non-normally distributed data were analysed by non-parametric testing. Categorical variables were assessed by the Chi-square test or Fisher's exact tests, as appropriate. Correlations were determined using Pearson's correlation coefficient (normally distributed) or Spearman's correlation coefficient (nonparametric data). A *P*-value < 0.05 was considered significant for all statistical testing while 0.05 < *P* < 0.1 was borderline significant.
